# Outcomes of Fenestration of Lamina Terminalis for Hydrocephalus following Clipping of Ruptured Aneurysms of Anterior Circulation

**DOI:** 10.12669/pjms.40.12(PINS).11122

**Published:** 2024-12

**Authors:** Ismaeel Khalid Iqbal, Nasruddin Ansari, Muhammad Subhan, Faiq Sheikh, Ijaz Hussain Wadd, Hafiz Abdul Majid

**Affiliations:** 1 Dr. Ismaeel Khalid Iqbal, MBBS, Department of Neurosurgery Unit-III, Punjab Institute of Neurosciences, Lahore, Pakistan; 2 Dr. Nasruddin Ansari, MBBS., Department of Neurosurgery Unit-III, Punjab Institute of Neurosciences, Lahore, Pakistan; 3 Dr. Muhammad Subhan, MBBS, Department of Neurosurgery Unit-III, Punjab Institute of Neurosciences, Lahore, Pakistan; 4 Dr. Faiq Sheikh, MBBS, FCPS(Neurosurgery), Department of Neurosurgery Unit-III, Punjab Institute of Neurosciences, Lahore, Pakistan; 5 Dr. Ijaz Hussain Wadd, MBBS, FCPS(Neurosurgery), Department of Neurosurgery Unit-III, Punjab Institute of Neurosciences, Lahore, Pakistan; 6 Prof. Dr. Hafiz Abdul Majid, MBBS, FCPS(Neurosurgery), Department of Neurosurgery Unit-III, Punjab Institute of Neurosciences, Lahore, Pakistan

**Keywords:** Lamina terminalis, Aneurysmal subarachnoid hemorrhage, Hydrocephalus, Cerebral aneurysms

## Abstract

**Objectives:**

To evaluate the Outcomes of Fenestration of Lamina Terminalis for Hydrocephalus following Clipping of Ruptured Aneurysms of Anterior Circulation.

**Methods:**

A retrospective case series study was conducted at the Punjab Institute of Neurosciences, Lahore from August 2022 to July 2023. Seventy seven patients meeting the inclusion criteria of age group 20-65 years, ruptured saccular aneurysm of anterior circulation with or without lamina terminalis fenestration during clipping, were enrolled through non-probability convenience sampling. Data was collected on all patients concerning postoperative hydrocephalus and the measures to mitigate its adverse effects. SPSS version 21 was utilized for data analysis.

**Results:**

The average age of the patients was 46, and 60% were female. In the Lamina terminalis fenestrated group (n=40), the percentage of shunt-dependent hydrocephalus cases following aneurysm clipping was 4% for low Fisher grade and 13% for high Fisher grade. In contrast, in the non-fenestrated group of (n=37) patients, the percentage was 10% for the low Fisher grade and 37% for the high Fisher grade.

**Conclusion:**

Reducing the likelihood of shunt-dependent hydrocephalus after ruptured aneurysm clipping can be achieved using lamina terminalis fenestration, regardless of fisher grade. It assists in preventing excessive brain retraction, decreasing intracranial pressure, raising brain perfusion, cisternal blood cleansing, and lowering the need for a ventriculoperitoneal shunt in cases of persistent hydrocephalus. Consequently, to reduce the incidence of shunt-dependent hydrocephalus, we recommend routine lamina terminalis fenestration following anterior circulation aneurysm clipping.

## INTRODUCTION

Blood in the subarachnoid and cisternal spaces is termed as subarachnoid hemorrhage. The global incidence of ruptured cerebral aneurysmal subarachnoid hemorrhage (aSAH) is 9/100000/year. However, in the United States, this fluctuates between 6-16/100000/year in various states, and it can reach up to 21.8/100000/year, which deviates slightly from the available international statistics.^1^ Acute subarachnoid hemorrhage is a life-threatening condition; 15% of the patients suffering from it die at the time of bleed, and 40% within a month.^2^ The management principle for a ruptured intracranial aneurysm is to completely occlude the Aneurysm either by microsurgical clipping or endovascular coiling and maintain the normal patency and circulation within the vessel.^3^

Ruptured aneurysmal bleed can be complicated by hydrocephalus, vasospasm, rebleed, and seizures. Hydrocephalus is very common following subarachnoid hemorrhage with an incidence of 6.5-67%, 6-45% of it requiring Cerebrospinal fluid diversion permanently. Hydrocephalus developing within three days of rupture of intracranial aneurysm is acute, 4-13 days is subacute, and after 14 days is chronic.^4^ Cerebrospinal fluid dynamics change after Subarachnoid hemorrhage, arachnoid granulations blockage by blood, and ventricular system adhesions lead to communicating type of hydrocephalus. Shunt-dependent hydrocephalus is defined as Enlarged ventricles of the brain causing deterioration in the neurology of the patient characterized by headache, vomiting, altered sensorium, urinary incontinence, gait ataxia, seizures, any motor or sensory deficit which needs permanent Ventriculoperitoneal shunt placement. Its pathogenesis is multifactorial, comprising multiple cellular signaling pathways and inflammatory processes.^5^

Acute hydrocephalus is treated postoperatively with serial Lumbar punctures or External Ventricular drainage, and chronic hydrocephalus via a Ventriculoperitoneal shunt. Insertion of Extra ventricular drain and fenestration of lamina terminalis is performed per-operatively for cerebrospinal fluid diversion.^6^

Lamina terminalis fenestration allows the brain to relax via CSF diversion to basal cisterns from ventricles, along with drainage of intraventricular blood, if any, and decreasing the intracranial pressure, resulting in improved cerebral perfusion. Various meta-analysis of the incidence of hydrocephalus following lamina terminalis fenestration during clipping of anterior circulation aneurysm and found it compelling. However, the efficacy of Lamina terminalis fenestration remained controversial. A randomized control trial on 50 patients conducted by Masoud Hatefi et al showed no significant difference.^7^ Our study aims to observe the efficacy of lamina terminalis fenestration in decreasing the incidence of shunt-dependent hydrocephalus following clipping of anterior circulation aneurysm.

## METHODS

A retrospective case series study was performed at the Punjab Institute of Neurosciences Lahore from August 2022 to July 2023. A total of 77 patients of spontaneous subarachnoid hemorrhage diagnosed and managed at the Punjab Institute of Neurosciences who underwent clipping of anterior circulation aneurysm with or without lamina terminalis fenestration were enrolled through non-probability convenience sampling meeting the predefined inclusion criterion.

Vital signs and neurology were recorded at the time of presentation. Medical management included maintenance of hydration, pain management, antiepileptic drugs and calcium channel blockers, laxatives, and nebulization. CT scan of the brain was followed by CT angiography of the brain, and Digital subtraction angiography was performed to confirm the diagnosis and surgical planning. Surgery was planned after reviewing these imaging studies. Every patient in this study was operated on after 14 days of subarachnoid bleeding. Controlled External ventricular drain was inserted in patients with preoperative hydrocephalus. Forty patients underwent lamina terminalis fenestration during clipping of anterior circulation aneurysm by a single chief Neurosurgeon, and 37 patients did not undergo lamina terminalis fenestration while clipping, which another chief Neurosurgeon performed. External Ventricular drain was clamped postoperatively in patients with preoperative hydrocephalus and followed with postoperative CT brain; if neurological deterioration or ventricular enlargement was observed, serial lumbar drainage followed by Ventriculoperitoneal shunting was performed if no clinical improvement was observed. Patients were followed up at six months with a plain brain CT scan.

Using SPSS 21, the collected data was analyzed. Mean and standard deviation were calculated for continuous variables such as age and categorical variables for instance gender, and post-operative complications were expressed in the form of frequencies and percentages.

### Inclusion criteria:

• Patients of both genders with the age group of (20-65) years with saccular aneurysm of the anterior circulation, having post-bleed days of more than 14 days that underwent clipping with or without lamina terminalis fenestration.

### Exclusion criteria:

• Patients with fusiform aneurysm of the anterior circulation, those who underwent endovascular coiling of the anterior circulation aneurysm, and patients with multiple comorbidities (Uncontrolled Hypertension, Uncontrolled Diabetes Mellitus, Ischemic heart disease) and bleeding diathesis.

### Surgical technique:

All the patients underwent clipping of anterior circulation aneurysms via standard Subfrontal or trans-Sylvian approach. Meticulous opening of the carotid-optic cistern was done, with drainage of CSF and subarachnoid blood, if any. Gentle vascular dissection from the Optic nerve followed until the Optic Chiasm was performed to visualize the lamina terminalis. A midline incision was given in the lamina terminalis in an avascular plane wide enough to drain the CSF and clots in interpeduncular CSF spaces.

### Consent and Ethics permission:

This was a descriptive case series study and presented no harm to the subjects studied. Exemption letter from Institutional review board was obtained with reference number 1779/IRB/PINS/APPROVAL/2024.

**Fig.1:**
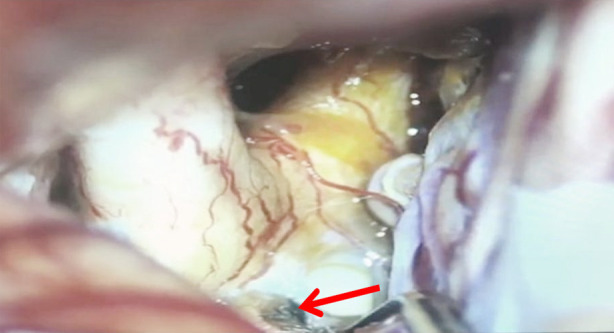
View of lamina terminalis fenestration during clipping of aneurysm.

### Operational definition:

***Hydrocephalus*** is a condition in which excessive cerebrospinal fluid builds up within the brain’s ventricles, leading to ventricle enlargement.

***Shunt-dependent hydrocephalus*** is characterized by enlarged ventricles of the brain, causing deterioration in the patient’s neurology. It is characterized by headache, vomiting, altered sensorium, urinary incontinence, gait ataxia, seizures, or any motor or sensory deficit that needs permanent Ventriculoperitoneal shunt placement.

## RESULTS

Seventy-seven patients were retrospectively reviewed for shunt-dependent hydrocephalus. Forty underwent Lamina terminalis fenestration, and 37 did not undergo fenestration following anterior circulation aneurysm clipping. Most of them were females (47), with an average age of presentation of 45.05 + 10.70 years (Mean ± SD). Details are shown in Table-I.

**Table-I:** Demographic characteristics.

Age Groups (Years)	Number of Patients
20-40	26
40-60	36
>60	15

*Gender*	*Number of Patients*

Male	33
Female	44

At the time of presentation, clinical vasospasm, despite adequate medical management, was seen in 12 patients (eight females and four males). Six patients underwent controlled external ventricular drain insertion for preoperative hydrocephalus, and four of them needed permanent Ventriculoperitoneal shunt.

Patients with Fisher grade 1-2 had a 10% risk of VPS who did not undergo LTF and 4% in those who had LTF following clipping of anterior circulation aneurysm. Similarly, those patients with high Fisher grade (3-4) had a 29.4% chance of chronic hydrocephalus requiring VPS, which was reduced to half, 13.3%, when it was clipped with lamina fenestration.

Four patients with Fisher grade 1-2 who underwent LTF developed hydrocephalus post-operatively and underwent serial lumbar puncture with drainage of 20-30 ml cerebrospinal fluid showed resolution of hydrocephalus. Details are shown in Table-II.

**Table-II:** Shunt-dependent hydrocephalus outcome analysis in LTF and Non-LTF patients post anterior circulation aneurysm clipping.

Fisher grade	LTF	Non-LTF	LTF-Shunt-dependent hydrocephalus (Percentage)	Non-LTF-Shunt-dependent hydrocephalus (Percentage)
1-2	25	20	1 (4.0 %)	2 (10.0 %)
3-4	15	17	2 (13.3%)	5 (29.4 %)

## DISCUSSION

Hydrocephalus is a frequent and fatal sequela of spontaneous SAH if not managed emergently. Bhattacharjee et al., in a detailed literature review documented a 6-67% risk of HCP post-SAH requiring short- or long-term CSF diversion.^8^ Meta-analyses of 15 pieces of literature with 2839 patients conducted at Chongqing Medical University by Mao J et al. and team exploring the advantage of Lamina terminalis fenestration during anterior circulation aneurysm clipping revealed a reduction in the incidence of shunt-dependent hydrocephalus. The fenestrated group had an 11.4% risk of hydrocephalus compared to the non-fenestrated group, which had a 15.3% risk with a relative risk reduction of 0.67 (95% confidence interval 0.50-0.90).^9^ Matinez-Perez et al., wrote to the editor in 2019 agreeing with Mao J et al., results. His prospective non-randomized study showed a 7.1% decreased need for a Ventriculoperitoneal shunt with a 3.9% absolute risk reduction. This was because of the wide cisternal opening, irrigation with 2L normal saline, and diluted papaverine.^10^ Similarly, Winkler et al. found reduced chronic hydrocephalus to as low as 5% via tandem lamina terminalis fenestration and Liliequist membrane fenestration following anterior circulation aneurysm clipping.^11^ Our study has a similar incidence of chronic hydrocephalus in patients with Fisher grade 1-2 that needed VP shunt post clipping of anterior circulation aneurysm (4%t), and those with high Fisher grade (3 or more) had 13% need for VPS.

Komotar et al., in various articles, had varied opinions regarding lamina terminalis fenestration. His first article, published in Neurosurgery 2002, supported the efficacy of lamina terminalis fenestration.^12^ However, a retrospective clinical series published in 2008 showed inconclusive benefits of LTF.^13^ Third article, where they did a systemic review of articles between 1950-2007 via MEDLINE search and gave negative opinions on microsurgical clipping, chronic hydrocephalus prevention, and reduction in vasospasm.^14^ Similarly, Ibrahim et al., published an article in neurosurgery concluding the null benefit of the method of aneurysm surgery in clearing clots from cisternal spaces after subarachnoid hemorrhage.^15^ However, Mura J et al., in their prospective non-randomized study for modified fisher grade scores of three or more, showed a reduction in the need for VPS to 3.2%.^16^

Our study found that high-grade SAH (fisher Grade-3 or more) had a higher chance of chronic hydrocephalus, similar to the one reported by Michael k. Tso et al., in 2016, who needed a permanent Ventriculoperitoneal shunt.^17^ Lamina terminalis fenestration reduced the need for VPS to almost 50% following aneurysm clipping, irrespective of fisher grade. Therefore, we advise developing a proper protocol of cisternal cleansing along with LTF in microsurgical clipping of anterior circulation aneurysms to reduce chronic hydrocephalus.

### Limitations:

The small sample size and uneven patient distribution may limit the generalizability of our findings. Additionally, the descriptive case series design does not allow for causal inferences. Therefore, a larger, multicenter, randomized, prospective study of longer duration is needed to confirm and expand upon our results.

## CONCLUSION

Lamina Terminalis fenestration is an effective procedure for reducing the risk of shunt-dependent hydrocephalus following clipping of ruptured aneurysm irrespective of fisher grade. It helps in brain relaxation, reducing intracranial pressure, increasing brain perfusion, cisternal cleaning of blood, preventing excessive brain retraction, and the need for a Ventriculoperitoneal shunt for chronic hydrocephalus. Therefore, we advocate regular lamina terminalis fenestration during anterior circulation aneurysm clipping to save as many patients as possible from a lifelong foreign body, i.e., Ventriculoperitoneal shunting dependent.

### Authors Contribution:

**IK** conceived, designed, did statistical analysis and editing of manuscript and is responsible for integrity of research.

**NA and MS** did data collection, analysis and manuscript writing.

**FS** did literature search, critical review and editing of manuscript.

**IW** did review and final approval of manuscript.

**AM** supervised the whole project and helped in preparation of manuscript.
